# The 5′ Leader of the mRNA Encoding the Mouse Neurotrophin Receptor TrkB Contains Two Internal Ribosomal Entry Sites that Are Differentially Regulated

**DOI:** 10.1371/journal.pone.0003242

**Published:** 2008-09-19

**Authors:** Stephanie L. Timmerman, Jennifer S. Pfingsten, Jeffrey S. Kieft, Les A. Krushel

**Affiliations:** 1 Department of Biochemistry and Molecular Genetics, University of Colorado Denver School of Medicine, Aurora, Colorado, United States of America; 2 Department of Pharmacology, University of Colorado Denver School of Medicine, Aurora, Colorado, United States of America; Lehigh University, United States of America

## Abstract

A single internal ribosomal entry site (IRES) in conjunction with IRES transactivating factors (ITAFs) is sufficient to recruit the translational machinery to a eukaryotic mRNA independent of the cap structure. However, we demonstrate that the mouse TrkB mRNA contains two independent IRESes. The mouse TrkB mRNA consists of one of two 5′ leaders (1428 nt and 448 nt), both of which include the common 3′ exon (Ex2, 344 nt). Dicistronic RNA transfections and *in vitro* translation of monocistronic RNA demonstrated that both full-length 5′ leaders, as well as Ex2, exhibit IRES activity indicating the IRES is located within Ex2. Additional analysis of the upstream sequences demonstrated that the first 260 nt of exon 1 (Ex1a) also contains an IRES. Dicistronic RNA transfections into SH-SY5Y cells showed the Ex1a IRES is constitutively active. However, the Ex2 IRES is only active in response to retinoic acid induced neural differentiation, a state which correlates with the synthesis of the ITAF polypyrimidine tract binding protein (PTB1). Correspondingly, addition or knock-down of PTB1 altered Ex2, but not Ex1a IRES activity *in vitro* and *ex vivo*, respectively. These results demonstrate that the two functionally independent IRESes within the mouse TrkB 5′ leader are differentially regulated, in part by PTB1.

## Introduction

In eukaryotes, recruitment of canonical factors including the ribosome to the 7-methyl guanosine cap structure on the 5′ end of the mRNA is thought to be the primary mechanism by which translation initiation occurs[1]. Once recruited to the mRNA, the 40S ribosome scans along the 5′ leader until it encounters the initiator codon [1]. However, initiation of a number of eukaryotic mRNAs can also occur through internal ribosomal entry sites (IRESes, for review see [2]), a mechanism first identified in viral RNAs [3]. In a manner analogous to the cap structure, a single IRES recruits the 40S ribosomal subunit to the mRNA for translation [4], [5].

Unlike initiation from the cap structure, the subset of proteins required for initiation from a eukaryotic IRES has yet to be determined. However, the current literature suggests that most eukaryotic IRESes require non-canonical translation factors referred to as IRES transactivating factors (ITAFs) for initiation. The three isoforms of polypyrimidine tract binding protein (PTB1, 2 and 4) [6] [7], La protein[8], and upstream of n-ras protein (unr) [9] are examples of ITAFs required for efficient translation from eukaryotic and viral IRESes. These proteins may function as chaperones, altering or maintaining specific RNA structures that permit binding of the translational machinery (for review see [10]). This theory is supported by work from the Niepmann lab demonstrating that binding of PTB to the foot-and-mouth disease (FMDV) IRES enhances the recruitment of the initiation factor eIF4G and stimulates FMDV IRES activity [11]. Additionally, it has been shown that the eukaryotic Apaf-1 5′ leader undergoes secondary structure changes upon the binding of unr and PTB, allowing for internal initiation of translation [5].

Utilization of eukaryotic IRESes often occurs during cellular events that reduce cap-dependent translation. For example, during mitosis the family of eIF4E binding proteins (4E-BP) is hypophosphorylated, which enhances their ability to bind eIF4E [12]. The sequestering of eIF4E inhibits cap-dependent translation [3] and the overall level of protein synthesis is decreased [13]. However, during mitosis several mRNAs, including those encoding for ornithine decarboxylase (ODC) [12] and p58 (PITSLRE kinase) [14], are translated in an IRES-dependent manner. For PITSLRE kinase, its IRES activity partially depends on the upregulation of unr expression at the G2/M junction underscoring not only the importance of the cell state, but also the importance of ITAF availability for internal initiation.

Studies in model systems indicate that neural activity may also promote dependency on internal initiation of translation. In response to activity-dependent neural plasticity, Aplysia neuroendocrine cells undergo a switch from cap-dependent to cap-independent translation [15]. Additionally, multiple dendritically localized mRNAs exhibit IRES activity within neural cell lines [16] and primary neuronal cultures [17] suggesting they may be translated in response to neural activity.

The mRNA encoding for the neurotrophin receptor TrkB is dendritically-localized [18] and encodes for a tyrosine kinase receptor that binds brain-derived neurotrophin factor (BDNF) (for review see [19]). The TrkB receptor is synthesized under diverse conditions and its activity contributes to multiple cellular processes (for review see [20]). In neural stem cells, the TrkB mediated pathway promotes cell proliferation [21]. In the adult, TrkB activity contributes to changes in synaptic efficacy and local connectivity, ultimately affecting learning and memory (for review see [19]). Owing to its diverse functions, TrkB expression is regulated at multiple steps in response to various cellular conditions [22], [23], [24]. For example, ischemia stimulates TrkB protein synthesis in order to promote cell survival [25]. Interestingly, ischemia also inhibits cap-dependent translation [26] suggesting that the upregulation of TrkB occurs in a cap-independent manner. In accordance with this observation, our lab has demonstrated that the 5′ leader of the mRNA encoding for human TrkB contains an IRES [16].

We hypothesized that if IRES-dependent translation was a critical mechanism for the synthesis of TrkB protein, then both the presence of an IRES and its mechanism would be evolutionarily conserved. The human TrkB 5′ leader is derived from alternative transcriptional start sites and alternative splicing of five exons [27]. Interestingly, the IRES was localized to exon 5, which is present in all 5′ leader variants [16]. Unlike the human 5′ leader, the mouse TrkB 5′ leader is transcribed from two promoters and is comprised of two exons [28]. However, similar to the human TrkB 5′ leader, all of the mouse TrkB 5′ leaders contain a 3′ common exon, exon 2. The human exon 5 and the mouse exon 2 sequences share a 63% identity with regions of high similarity on the extreme ends of the exons. Consequently, we predicted that the mouse TrkB 5′ leader would mediate internal initiation and that the IRES would be located within Exon 2.

In the present report we establish that the mouse TrkB Exon 2 does contain an IRES. But surprisingly, an IRES is also located within Exon 1 demonstrating the presence of two IRESes initiating translation of the same open reading frame. The two IRESes are differentially utilized based on the differentiation state of a neuronal cell line. This phenomenon is explained in part by the observation that PTB1 differentially affects translation mediated by the two IRESes, despite the fact that PTB1 binds both IRESes with similar affinity.

## Results

### The Mouse TrkB 5′ Leaders Contain a Potential IRES Element

**Figure 1:**
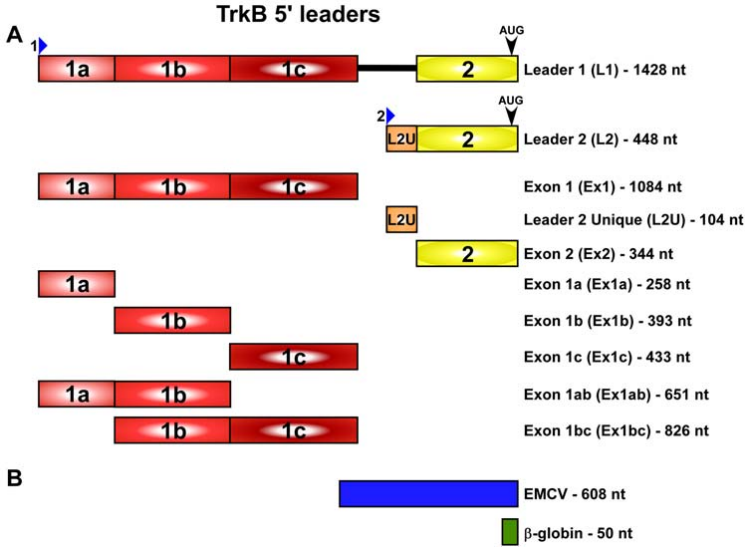
The mouse TrkB 5′ leaders. (A) Schematic representation of the gene structure of the mouse TrkB 5′ leader [28] and variations used for luciferase assays (B) as well as the two controls, the β-globin 5′ leader and EMCV IRES. The boxes represent exons, lines represent introns, and the blue arrows indicate the transcriptional start site of the two promoters.

To determine if the full-length 5′ leaders generated from the first promoter, Leader 1 (L1, 1.428 kb), from the second promoter, Leader 2 (L2, 448 nt), and Exon 2 (Ex2, 344 nt) exhibited IRES activity, they were inserted into the intercistronic region of the dicistronic luciferase DNA vector pRF (Fig 1a and 1b)[29]. The 5′ leader of the human β-globin mRNA (50 nt) and the leader of the encephlomyocarditis virus (EMCV) (608 nt) were also inserted as negative and positive controls, respectively [30]. The individual constructs were transfected into two neural cell lines, C6 and N2a, and after 24 hours the cells were harvested and assayed for luciferase activity. A ratio of the *Photinus* luciferase to the *Renilla* luciferase was calculated following a luciferase assay of the cell lysates. The ratio obtained for the negative control, β-globin, was set to one, and the ratios obtained from the other constructs were normalized to that value.

**Figure 2:**
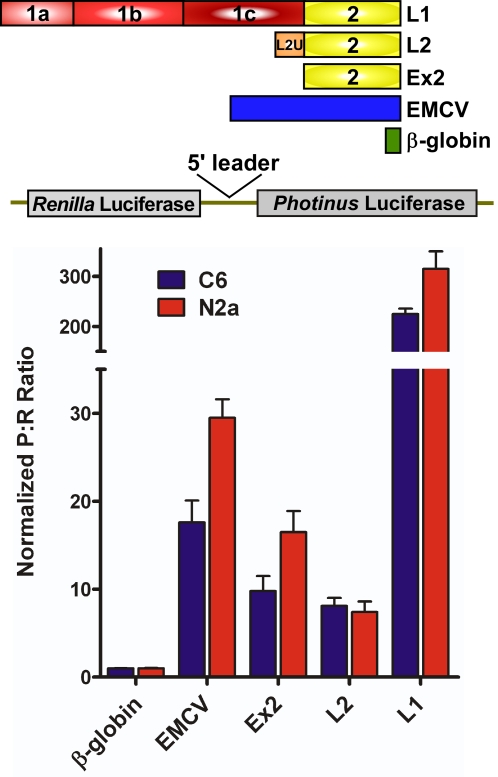
The mouse TrkB 5′ leaders exhibit an increased *Photinus* to *Renilla* luciferase (P∶R) ratio. Dicistronic luciferase constructs containing the β-globin, EMCV, and TrkB 5′ leaders were transfected into the C6 and N2a neural cell lines. The P∶R ratio from each construct was normalized to that obtained from the β-globin construct, whose P∶R ratio was set to one.

As expected, the positive control EMCV exhibited an increased *Photinus*:*Renilla* (P∶R) luciferase ratio when compared to the β-globin 5′ leader in both cell lines. EMCV generated a ratio of 28 in C6 cells and 16 in N2a cells (Fig 2). All three mouse TrkB 5′ leaders also exhibited higher P:R ratios than the negative control. Ex2 exhibited a P∶R ratio of 9 in C6 cells and 15 in N2a cells, while the full-length L2 had a ratio of 7 in both cell lines. Interestingly, L1 yielded a ratio ranging from 200–300 in the cell lines. These results suggest that all three mouse TrkB 5′ leaders can internally initiate translation, however the presence of other regulatory elements could also lead to an increased P∶R ratio from the DNA construct.

### A Cryptic Promoter is Present in the Mouse TrkB 5′ Leader

The dicistronic DNA construct has been used as the major assay to test for IRES activity. However, additional mechanisms can account for translation of the second cistron. The dicistronic DNA construct used in these assays contains an intron upstream of the *Renilla* luciferase gene to increase transcription and translation efficiency. However, the presence of a cryptic splice site located in the 5′ leader of interest would lead to the splicing of the *Renilla* luciferase gene, leaving only the *Photinus* luciferase gene to be translated and effectively increasing the P∶R ratio. In addition, the presence of a cryptic promoter in the 5′ leader would generate a monocistronic mRNA encoding only the *Photinus* luciferase gene. This process would create an mRNA consisting only of the *Photinus* luciferase gene and again, would lead to an increase in the P∶R ratio.

**Figure 3:**
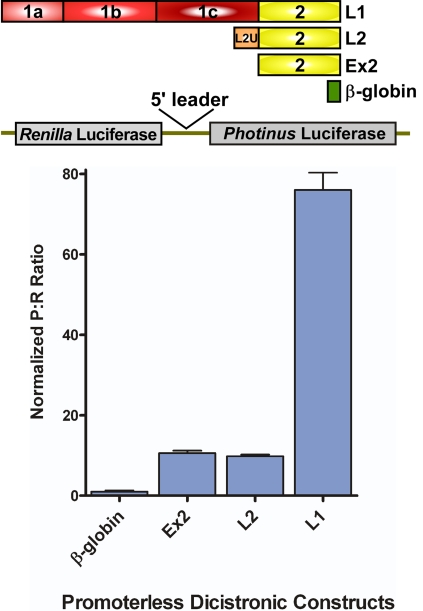
The mouse TrkB 5′ leaders exhibit cryptic promoter activity. Promoterless dicistronic luciferase constructs containing the β-globin or the TrkB 5′ leaders were transfected into C6 cells. The P∶R ratio was normalized to that obtained from construct containing the β-globin 5′ leader.

To determine if a cryptic promoter is present in the TrkB 5′ leaders, we inserted the TrkB and the β-globin 5′ leaders into the intercistronic region of a promoterless dicistronic luciferase construct (a generous gift from Dr. Anne Willis [31]). Transfection of the promoterless dicistronic DNA into C6 cells yielded robust *Photinus* luciferase activity from the TrkB L1 5′ leader (Fig. 3); the P∶R ratio was 76 fold higher than that obtained from the dicistronic construct containing the β-globin 5′ leader. In addition, both Ex2 and L2 showed a ten-fold increase in the P∶R ratio. It was perhaps surprising that L2 and Ex2 exhibited cryptic promoter activity because a Northern blot analysis did not reveal additional RNA species (data not shown), although this incongruity has been observed previously [32]. These results indicate that all three TrkB 5′ leaders exhibit some level of cryptic promoter activity.

### The Mouse TrkB 5′ Leader Internally Initiates Translation from Dicistronic RNA

**Figure 4:**
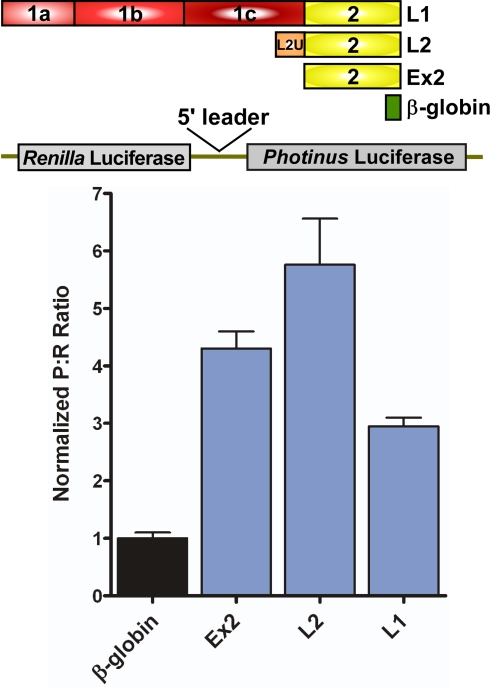
The mouse TrkB 5′ leaders exhibit IRES activity when expressed in dicistronic RNA constructs. Dicistronic luciferase mRNA containing the β-globin and the TrkB 5′ leaders were transfected into C6 cells. The P∶R ratio for each construct was normalized to that of the negative control, β-globin.

To overcome the limitations of cryptic promoter activity (and cryptic splicing which we did not examine), we *in vitro* transcribed the dicistronic DNA. The resulting dicistronic mRNA was transfected into C6 cells. All three TrkB 5′ leaders exhibited a P∶R ratio higher than that observed from the dicistronic mRNA containing the β-globin 5′ leader (Fig. 4). The largest ratio of approximately six was seen with L2. Ex2 generated a P∶R ratio of four, and L1 showed the lowest ratio of approximately 2.5. This result demonstrates that all three mouse TrkB 5′ leaders can mediate IRES-dependent translation. The result also indicates that the presence of a cryptic promoter in a 5′ leader does not preclude its ability to internally initiate translation.

### Mouse TrkB 5′ Leaders Initiate Translation When Cap-Dependent Translation is Inhibited

With a capped monocistronic RNA, it cannot be determined whether translation initiation is occurring in a cap-dependent manner or through an IRES. To dissociate between the two mechanisms, cap-dependent translation was inhibited *ex vivo*. We inserted the 5′ leaders upstream of the *Photinus* luciferase gene. The *Renilla* luciferase gene was contained on the same plasmid (pRM) under an independent promoter and served as an internal transfection control (Fig 1b). The multiple cloning site from the pGL3 plasmid (83 nt) was used as a negative control in this experiment because the Renilla gene on the pRM plasmid also utilizes this multiple cloning site as its 5′ leader. We have previously shown that the pGL3 multiple cloning site does not contain an IRES and exhibits a P∶R ratio equivalent to that obtained from a dicistronic DNA construct containing the β-globin 5′ leader [16]. The mouse TrkB and pGL3 DNA constructs were co-transfected into C6 cells with either a hypophosphorylated form of 4EBP [33] or a null vector. When expressed, the hypophosphorylated 4EBP sequesters eIF4E, inhibiting cap-dependent translation [34]. The percentage of luciferase activity remaining in the presence of the hypophosphorylated form of 4EBP when compared to that observed in the presence of the null vector was calculated. Translation of *Renilla* luciferase mRNA containing the pGL3 multiple cloning site served as the internal control to monitor cap-dependent translation.

**Figure 5:**
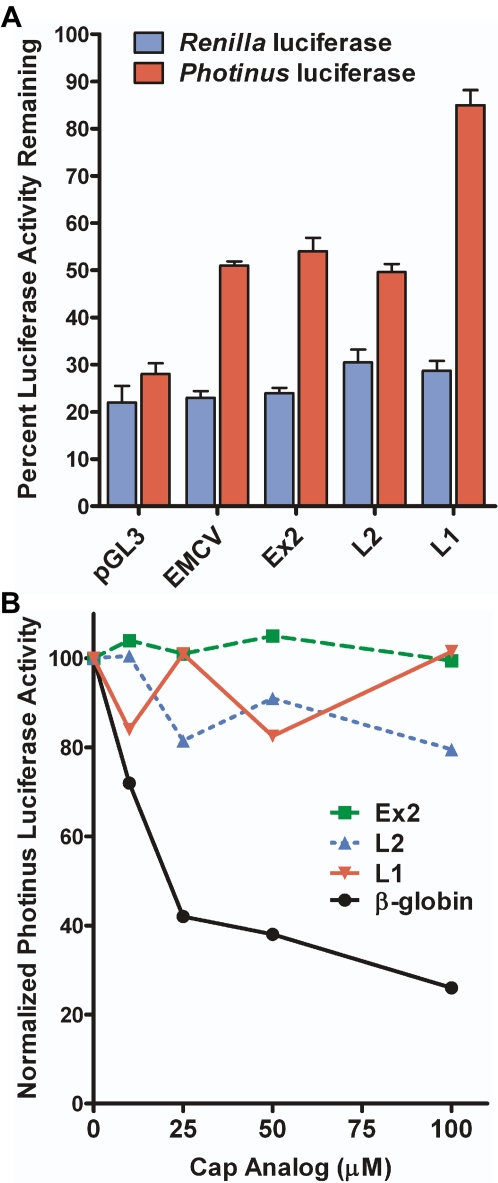
The mouse TrkB 5′ leaders are able to initiate translation when cap-dependent translation is inhibited. A) A dual monocistronic construct containing the pGL3 multiple cloning site upstream of the *Renilla* luciferase and the pGL3 multiple cloning site, EMCV, or the TrkB 5′ leaders upstream of the *Photinus* luciferase gene was co-transfected into C6 cells with a construct encoding for a hypophosphorylated 4EBP construct or a null vector. The level of *Photinus* luciferase activity from each mRNA, when co-transfected with the null vector, was normalized to 100 percent. The *Renilla* and *Photinus* luciferase activity obtained in cells co-transfected with hypophosphorylated 4EBP is represented as a percentage of the activity obtained in cells co-transfected with the null plasmid. B) Monocistronic *Photinus* luciferase mRNA containing the β-globin or TrkB 5′ leaders was *in vitro* translated in rabbit reticulocyte lysate in the presence of increasing concentrations of cap analog. *Photinus* luciferase activity for each mRNA in the absence of cap analog was set to 100 percent.

In the presence of hypophosphorylated 4EBP, the *Renilla* luciferase activity decreased to approximately 23–30% of the control level for all of the constructs. This result demonstrated that cap-dependent translation was being inhibited (Fig 5a). In addition, the level of *Photinus* luciferase activity generated from the mRNA containing the multiple cloning site from the vector pGL3 decreased to 28% of the control confirming that its translation was also cap-dependent. On the other hand, the *Photinus* luciferase mRNA containing the EMCV IRES decreased to 51% of the control transfection when cap-dependent translation was inhibited confirming that it does initiate translation through a cap-independent mechanism. The relatively large decrease in the overall level of translation from the mRNA containing the EMCV IRES when cap-dependent translation is shut down is believed to be due to the addition of a cap structure to the mRNA. This creates an artificial context for the EMCV 5′ leader and when uninhibited, allows for the cap structure to compete with the IRES for translational machinery. All three mRNAs containing the different mouse TrkB 5′ leaders showed a smaller reduction in activity compared to that observed from the pGL3 mRNA. Ex2 and L2 exhibited similar reductions of approximately 50%. L1 exhibited only a 15% decrease in *Photinus* luciferase activity in the presence of hypophosphorylated 4EBP. This result supports the conclusion that all three TrkB 5′ leaders can initiate translation independent of the cap. It also suggests that the contribution of cap- and IRES-dependent translation may vary for each leader.

To further demonstrate the ability of the TrkB 5′ leaders to initiate cap-independent translation, *in vitro* transcribed mRNA containing the three mouse TrkB 5′ leaders or the β-globin 5′ leader upstream of the *Photinus* luciferase open reading frame was translated in rabbit reticulocyte lysate (RRL). Increasing concentrations of cap analog, which binds and sequesters eIF4E inhibiting cap-dependent translation, was added to the lysate. Indeed, the level of *Photinus* luciferase activity derived from the mRNA containing the β-globin 5′ leader was inversely proportional to the concentration of cap analog (Fig 5b). However, the level of translation from the mRNAs containing the mouse TrkB 5′ leaders was relatively stable irrespective of the cap analog concentration. These results demonstrate that the mRNA containing the TrkB 5′ leader can be translated in a cap-independent manner.

### The Mouse TrkB 5′ Leader Contains Multiple Contains Multiple IRESes

**Figure 6:**
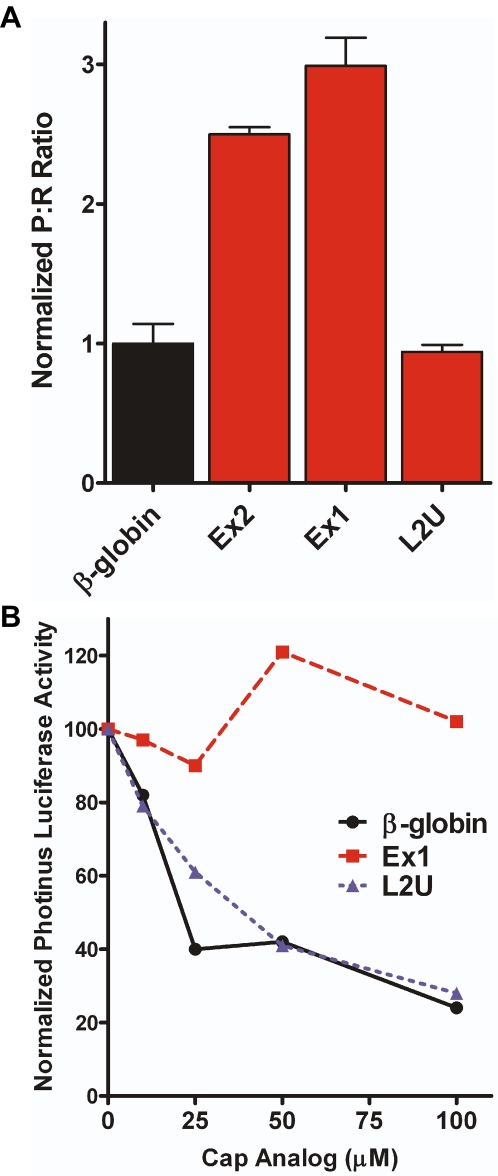
The mouse TrkB mRNA contains two independent IRESes. A) The additional sequence upstream of Exon 2 from both full-length leaders (Ex1 and L2U) and Exon 2 alone (see Fig 1a) were individually inserted into dicistronic RNA vectors and transfected into C6 cells. The resulting P∶R ratios were normalized to the ratio observed from the mRNA containing the β-globin 5′ leader. B) The unique regions of the full-length TrkB 5′ leaders, as well as the β-globin 5′ leader were inserted upstream of a monocistronic *Photinus* luciferase open reading frame and *in vitro* translated in the presence of increasing concentrations of cap analog. The initial level of *Photinus* luciferase activity was set to 100 percent for each mRNA.

Our results indicate that an IRES element is located within exon 2, which is present in both mouse TrkB 5′ leaders. To determine whether the unique upstream regions in each 5′ leader can also internally initiate translation, these regions were inserted into dicistronic RNA constructs and transfected into C6 cells. The unique region from Leader 2, L2U (104 nt, Fig 1a), generated a P∶R ratio similar to that observed with the negative control β-globin (Fig 6a). However, the upstream sequence from Leader 1, Ex1 (1.084 kb), generated a P∶R ratio comparable to that observed from Ex2 indicating that Ex1 contains an IRES.

To confirm the *ex vivo* results, we inserted Ex1 and Pr2U into monocistronic *Photinus* constructs and *in vitro* translated the mRNA (Fig 6b). As expected, translation from the L2U construct decreased in the presence of increasing amounts of cap analog similar to that observed from the mRNA containing the β-globin 5′ leader. On the other hand, translation from the Ex1 construct remained relatively constant. Taken together, the *ex vivo* and *in vitro* data demonstrate the ability of Ex1, in addition to Ex2, to internally initiate translation.

**Figure 7:**
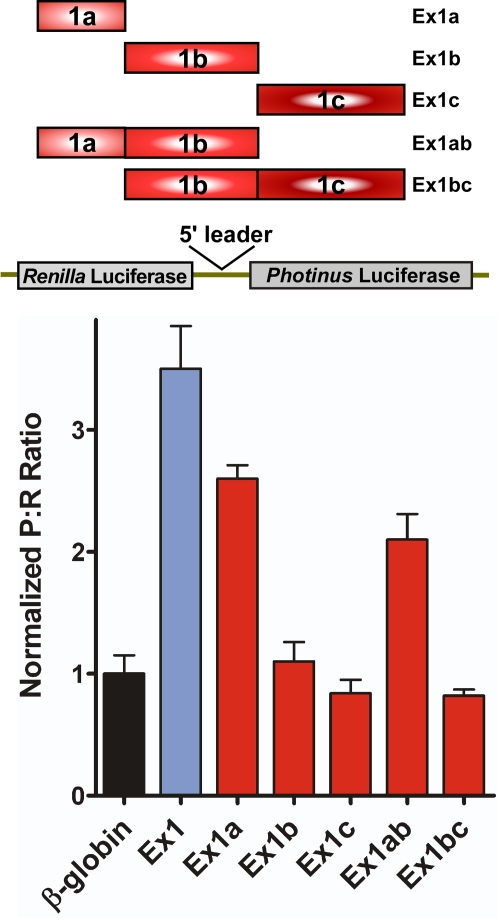
The upstream IRES in the mouse TrkB 5′ leader is located within Ex1a. The exon 1 splice variants (shown in the schematic, see also Fig 1A) were inserted into dicistronic RNA vectors and transfected into C6 cells. The resulting P∶R ratios were normalized to the ratio observed from β-globin 5′ leader.

Exon 1 of mouse TrkB can be alternatively spliced at three sites creating three different leaders, which all contain the first 259 nt of the exon (Fig 1a). To identify the region within Exon 1 that contains the IRES we created dicistronic constructs containing the individual segments, Ex1a, Ex1b, and Ex1c, or in contiguous pairs, Ex1ab and Ex1bc. Transfection of C6 cells with the dicistronic RNA showed that all 5′ leaders containing Ex1a generated a P∶R ratio higher than β-globin (Fig 7). However, Ex1b, Ex1c, and Ex1bc exhibited a P∶R ratio equivalent to that obtained from β-globin. These results indicate that the IRES element in Ex1 is located within Ex1a.

### PTB1 Protein Binds both Mouse TrkB IRESes

**Figure 8:**
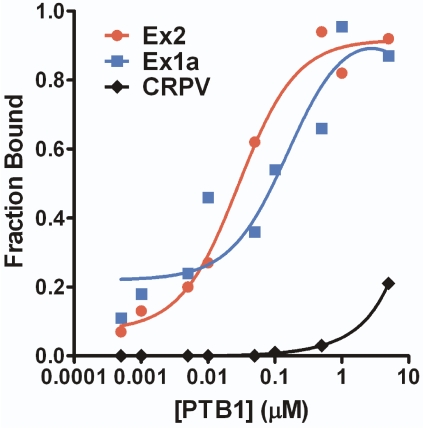
The mouse TrkB 5′ leaders bind PTB1 protein. Purified PTB1 was added in increasing amounts to radiolabeled RNA containing Ex1a, Ex2, or the negative control CrPV IRES. A Langmuir plot was created using the calculated fraction bound. Disassociation constants of 85 nM and 46 nM were determined for the PTB1 interaction with Ex1a and Ex2, respectively.

PTB activates or enhances IRES activity from a number of cellular mRNAs [6], [9], [35]. As mentioned previously, unr and PTB bind to the Apaf-1 IRES, inducing conformational changes and allowing for internal initiation to occur [5]. PTB binds to the Apaf-1 IRES at two polypyrimidine tracts located 74 and 118 nucleotides from the initiator codon, respectively. Sequence comparison revealed that the mouse TrkB 5′ leader has two polypyrimidine tracts in similar locations of 75 and 124 nucleotides from the initiaton codon suggesting that PTB may also influence RNA secondary structure, and ultimately the IRES activity, of the mouse TrkB 5′ leader. To determine the ability of PTB1 to directly bind the Ex1a and Ex2 IRESes, we performed a filter binding assay. The cricket paralysis virus (CrPV) IRES was used as a negative control since it has been established that the CrPV IRES does not require protein factors to initiate translation [36]. Radiolabeled RNA consisting of Ex1a, Ex2, or CrPV was incubated in the presence of increasing amounts of recombinant PTB1 protein and passed through a dot blot apparatus. The resulting binding curve was fit using the Langmuir equation. As expected, the CrPV IRES did not bind to PTB1 with a significant affinity (Fig 8). However, Ex1a and Ex2 bound to PTB1 with K_d_ values equaling 85 nM and 46 nM, respectively. Although PTB1 binding to both IRESes falls within the same order of magnitude, the two-fold difference may reflect a biologically significant difference.

### The Two TrkB IRESes Are Differentially Regulated

**Figure 9:**
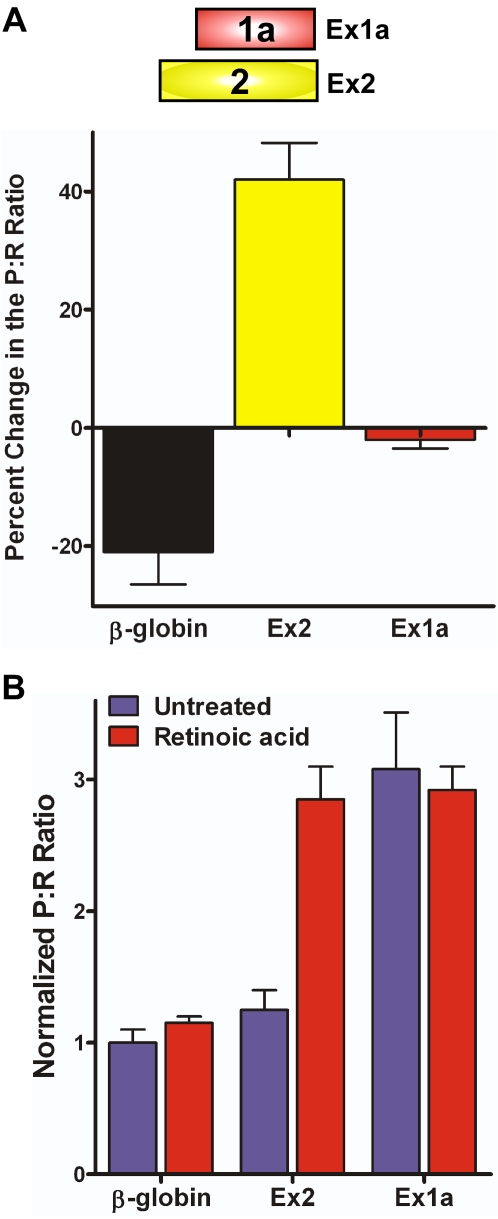
PTB increases IRES activity from the Ex2 IRES, but does not affect Ex1a IRES activity. A) Dicistronic luciferase mRNA containing the Ex1a, Ex2, or β-globin 5′ leader was *in vitro* translated in RRL that was either untreated or supplemented with 0.4 mg of PTB1. The change in the P∶R ratio for the samples when in the presence of PTB1 relative to the untreated sample are shown. B) The two mouse TrkB IRESes demonstrate differential regulation. SH-SY5Y cells treated with 2 mM retinoic acid or with DMSO (mock) for four days were transfected with dicistronic mRNA containing the β-globin, Ex2, or Ex1a 5′ leaders. The P∶R ratios were normalized to that from the mRNA containing the β-globin 5′ leader.

To address whether the binding of PTB1 plays an important role in Ex1a and Ex2 IRES activity, dicistronic RNA was *in vitro* translated in the presence of 0.4 µg of PTB1 protein isoform [37]. Somewhat surprisingly, no change in the P∶R ratio from Ex1a was observed when PTB1 was present despite the ability of PTB1 to bind to Ex1a (Fig 9a). Conversely, the P∶R ratio from the dicistronic mRNA containing Ex2 increased by 40% in the presence of PTB1. This result suggests that PTB1 stimulates Ex2 IRES activity and that PTB1 interacts with the mouse TrkB IRESes differently.

Changes in cell state, such as mitosis [12], inhibit cap-dependent translation and promote ITAF synthesis and IRES activity. Since the TrkB receptor contributes to different cellular functions in neural stem cells and neurons, we were interested in determining whether differentiation also affected IRES activity. To examine the effects of differentiation on the TrkB IRESes, we chose SH-SY5Y cells, a neuroblastoma that generates a differentiated neuronal phenotype when exposed to retinoic acid [9]. Dicistronic RNA containing Ex1a, Ex2, or the β-globin 5′ leader was transfected into SH-SY5Y cells that were either treated with DMSO (undifferentiated) or retinoic acid (differentiated) for four days (Fig. 9b). The dicistronic mRNA containing Ex1a exhibited a P∶R ratio approximately three-fold higher than β-globin in both the undifferentiated and differentiated cells. Surprisingly, in undifferentiated cells Ex2 did not demonstrate IRES activity, yielding a P∶R ratio similar to that of β-globin. However, in the differentiated cells the P∶R ratio of Ex2 increased to approximately two and a half fold that of β-globin. This result suggests that the Ex1a IRES is constitutively active is SH-SY5Y cells, while the Ex2 IRES is only active in differentiated SH-SY5Y cells.

**Figure 10:**
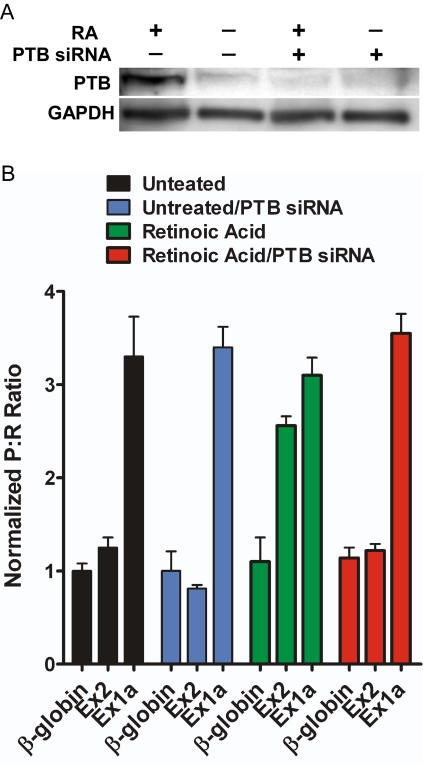
PTB expression is required for IRES activity mediated by Ex2. A) Western blot analysis of PTB and GAPDH (as a loading control) from lysates obtained from differentiated and undifferentiated SH-SY5Y cells transfected with siRNA directed against PTB or mock transfected for 72 hours. B) Dicistronic RNA containing the two mouse TrkB IRESes were transfected into differentiated SH-SY5Y cells depleted of PTB1 by siRNA. The P∶R ratios were normalized to that from the mRNA containing the β-globin 5′ leader in control undifferentiated SH-SY5Y cells.

The ability of Ex2 to internally initiate translation in differentiated but not undifferentiated SH-SY5Y cells indicates the presence of a factor that is induced upon differentiation. Since we have already established that PTB1 affects the IRES activity from the mouse TrkB IRESes differentially, it suggests that PTB1 may be the factor responsible for the differential regulation observed in the SH-SY5Y cells. Indeed, differentiation of SH-SY5Y cells by retinoic acid leads to the induction of PTB1 [9], but not the neural isoform, nPTB (data not shown). To investigate whether the presence of PTB1 affects TrkB IRES activity, PTB1 protein was knocked down in differentiated SH-SY5Y cells using siRNA. Western blot analysis revealed that the siRNA reduced PTB1 expression level by greater than 80% from that observed in untreated differentiated cells (Fig. 10a). Additionally, transfection of dicistronic RNA into the PTB1 depleted differentiated SH-SY5Y cells showed a decrease in Ex2 IRES activity, suggesting that PTB1 is required for internal initiation from the Ex2 IRES (Fig. 10b). The Ex1a IRES activity was not affected by the reduction in PTB1, confirming the differential regulation seen above.

## Discussion

In the present report, we demonstrate that the mouse TrkB 5′ leader contains two IRESes. One IRES is located within Ex2, a region similar to the region within the human TrkB 5′ leader found to exhibit IRES activity [16]. The other IRES is unique to the mouse TrkB 5′ leader and is found in the 5′ end of Exon 1. Moreover, we found that the two IRESes exhibit different characteristics. The IRES in Ex2 is active in differentiated SH-SY5Y cells, binds PTB1, and its activity is enhanced in the presence of PTB1. On the other hand, the IRES within Ex1a is active in both differentiated and undifferentiated SH-SY5Y cells, binds PTB1, but its activity is unaffected in the presence of PTB1. We also noted that the TrkB 5′ leaders contain a cryptic promoter, indicating that 5′ leaders can contain both a cryptic promoter, as well as an IRES.

### Limitations of Using Dicistronic DNA Constructs to Demonstrate IRES Activity

Historically, dicistronic constructs have been used to examine 5′ leaders for the ability to internally initiate translation. However, the increased P∶R ratios that have been interpreted as the presence of an IRES can also be due to the presence of cryptic splice sites or cryptic promoters [32]. Removal of the promoter and intron from the dicistronic construct demonstrated that all three TrkB 5′ leaders exhibit some level of cryptic promoter activity. This result does not rule out the possibility that cryptic splicing occurs in the original dicistronic vector, and therefore, it is still possible that both processing events can occur and contribute to the second RNA species.

Our results indicate that the presence of a cryptic promoter in a eukaryotic 5′ leader does not preclude the presence of an IRES. Indeed, it was previously reported that both an IRES and a cryptic promoter are present in the 5′ leader of hepatitis C virus (HCV) [38]. To more conclusively demonstrate IRES activity in cells, we transfected cells directly with RNA. This approach should reveal IRES activity exhibited by mRNA without subjecting it to nuclear processes. Indeed, aberrant RNA species following transfection of DNA constructs have not been observed when the corresponding RNA was transfected [39]. Using RNA transfections, all three mouse TrkB 5′ leaders demonstrated IRES activity. In addition, the TrkB 5′ leaders were able to initiate translation *in vitro* when cap-dependent translation was inhibited. Together, these data suggest that despite the presence of a cryptic promoter, the mouse TrkB 5′ leaders can internally initiate translation.

### Multiple IRESes Present in a Single mRNA

The mouse TrkB mRNA is one of only a few eukaryotic mRNAs to contain two IRESes within the same mRNA. Transcription of the mouse TrkB gene from promoter 1 yields the 5′ leader 1 with IRESes located within Ex1 and Ex2. The IRES in Ex1 was further localized to the 5′ end, a region that is within all alternatively spliced variants. The rationale for multiple IRESes on the same 5′ leader is not known. It is possible that multiple IRESes work cooperatively to increase IRES activity. If this was occurring with the Ex1a and Ex2 IRESes, we predict the presence of both IRESes (L1) would generate higher IRES activity than either IRES alone. However, L1 exhibited a lower P∶R ratio than either Ex1a or Ex2 alone (Fig 4).

A second possibility is that the two IRESes initiate at different initiator codons. This situation occurs with the two IRESes located within the c-myc mRNA. The upstream element, IRES 1, initiates translation of the MYCHEX1 open reading frame (ORF) while the downstream IRES 2 initiates the c-myc 1/ c-myc 2 ORF [40]. Additionally, two IRESes exist in the 5′ leader of the mRNA coding for endothelial growth factor (VEGF). They control initiation at two alternative start sites yielding a protein with a different N-terminus [41], [42]. Interestingly, an upstream ORF (uORF) located between the two IRESes determines which IRES is utilized [43]. The mouse TrkB Exon 1 contains 15 potential uORFs that could also regulate the usage of the two TrkB IRESes. Only one uORF (−1048 nt in Ex1b) is in a moderately favorable Kozak context. It would therefore, be of interest to determine if the predicted 4.2 kD product encoded in Exon 1 is synthesized. We cannot identify at present, whether one or both IRESes is utilized in the full length L1 5′ leader. However, since all 5′ leaders containing one or two IRESes (regardless of the presence of uORFs) yield equivalent levels of luciferase protein it indicates that the major ORF is the *Photinus* luciferase ORF. This observation is in agreement with the identification of a single initiation start site in both the mouse and human TrkB genes [27], [28], and would be the first example of two unique IRESes being used to produce the identical protein.

A third explanation is that the two IRESes are differentially employed. Multiple mechanisms may exist to ensure the presence and/or use of one IRES. For example, neural activity increases transcription mediated by promoter 2 generating an mRNA with only one IRES [44]. Second, the presence of RNA upstream of an IRES may inhibit its function [45]. In this case, the Ex1a IRES would be active in the full-length leader if the presence of the upstream sequence inhibits the Ex2 IRES. Finally, when both IRESes are present, IRES selection may be regulated by the presence of ITAFs. It has been well documented that IRESes are neither constitutively nor ubiquitously active. IRESes, including those in the mouse TrkB 5′ leaders, as well as human TrkB [16], c-IAP1 [46], Apaf-1 [47] and c-myc [48], demonstrate varying activities in different cell lines, presumably due to differential expression of ITAFs. Indeed, we have shown that the two IRESes within the TrkB 5′ leader are differentially regulated within a cell line. This level of regulation is likely functional within primary neurons. For example, Ex2 IRES is only active in differentiated SH-SY5Y cells, a model for post-mitotic neurons that are capable of neural activity [49]. Neural activity in turn promotes the use of promoter 2 generating a TrkB 5′ leader containing only the Ex2 IRES [44]. This observation provides a link between the differential usage of the TrkB promoters and TrkB translation.

At the molecular level, differential usage of the two TrkB IRESes may be regulated in part by PTB1. IRES activity *in vitro* mediated by the Ex2 IRES was increased in the presence of PTB1. In addition, Ex2 IRES activity correlates with the expression pattern of PTB1 within SH-SY5Y cells [9] and is decreased when PTB1 levels are significantly reduced. IRES activity mediated by Ex2 mirrors that of the Apaf-1 IRES in that it is also only active in differentiated SH-SY5Y cells and is regulated by PTB1 [9].

Binding of an accessory protein to an mRNA does not implicate the protein as an ITAF. *In vitro* translation assays and RNA transfections into SH-SY5Y cells demonstrate that although PTB1 binds to both Ex1a and Ex2, it only affects Ex2 IRES activity. Ex1a does not require PTB1 to internally initiate translation and the changes in the level of PTB1 does not alter Ex1a IRES activity, an observation seen previously for one of the IRESes in the VEGF 5′ leader [42]. It remains possible that PTB1 plays a role in enhancing Ex1a IRES activity in conjunction with additional factors that are absent from RRL and SH-SY5Y cells. The Apaf-1 and BAG-1 IRESes require the presence of unr and poly(rC) binding protein 1 respectively, in addition to PTB1, for IRES activity [5], [50].

The position of the PTB1 site within the mRNA secondary structure can determine whether PTB1 binding can affect IRES activity. Therefore, the inability of PTB1 to enhance Ex1a IRES activity may be due to the context of the PTB1 binding sites. The Willis lab demonstrated that the PTB1 binding site, a CCU repeat, can only internally initiate translation when present within a stem structure and not as single stranded RNA [31]. Consequently, it would be predicted that potential PTB1 binding sites within Ex2 are in a double stranded conformation, while those within Ex1a would exist in a single stranded state.

It is also possible that PTB1 binding to the TrkB mRNA occurs to mediate another mRNA processing event. PTB1, in addition to regulating internal initiation, also affects RNA splicing [37]. Since Exon 1 is alternatively spliced, it is possible that PTB1 is binding for that purpose. In addition, the assay was performed in isolation of other polypyrimidine binding proteins that may normally compete with PTB1 for the binding sites within TrkB and prevent the binding of PTB1.

Differential use of the TrkB IRESes may be a mechanism to regulate protein expression levels. For instance, the selection of which IRES is utilized will alter the sequence and distance over which the ribosome must scan, in a similar manner to that predicted for ribosomes recruited to the cap structure. For example, a long G/C rich 5′ leader will impede ribosomal scanning and decrease the overall level of protein synthesis [51]. In the case of TrkB, the distance from the Ex1a IRES is over 1kb long, is G/C rich, and contains multiple upstream AUGs. Therefore, when less TrkB is required, initiation could occur from the Ex1a IRES. However, when TrkB upregulation is required to affect synaptic plasticity in response to neural activity, initiation could occur from the shorter, and now more prevalent Ex2 IRES. Consequently, the presence of multiple IRESes on a single 5′ leader may provide an additional mechanism to regulate protein synthesis.

## Materials and Methods

### Constructs

The mouse TrkB 5′ leaders (Fig 1a), with the exception of L1, were cloned from a mouse brain cDNA library (Clontech) with EcoRI and NcoI restriction sites. L1 was created by PCR amplifying exon 1 and exon 2 separately. Exon 1 was created with an EcoRI site at the 5′ end and a blunt 3′ end. Exon 2 had a blunt 5′ end and an NcoI site at its 3′ end. The central blunt ends were ligated together and the combined exons were the inserted into the RF vector using the EcoRI and NcoI sites at the insert ends. The RF vector was a generous gift from Dr. Anne Willis, University of Nottingham.

In order to create the dual monocistronic vector, the RF constructs were then digested with EcoRI and HpaI to isolate the 5′ leader, the *Photinus* luciferase gene, and the SV40 3′ UTR. This portion was then ligated into the pGL3 vector (Promega). To isolate the 5′ leader and *Photinus* luciferase gene from this vector, it was digested with SnaBI and ApaLI. The fragment was then ligated into a backbone that already contained a monocistronic *Renilla* luciferase gene, creating a vector that contains both luciferase genes in a monocistronic context. The mono- and dicistronic constructs used for *in vitro* transcription were made as described in [16].

To create the promoterless constructs, the β-globin RP vector was digested sequentially with SmaI and EcoRV to release the upstream intron and promoter. The blunt ends were then ligated together to create the promoterless vector. The 5′ leaders were exchanged using the EcoRI and NcoI restriction sites.

DNA containing the cricket paralysis virus (CrPV) IRES was PCR amplified from plasmid DNA designed as described in [52] using M13 primers. The original CrPV IRES DNA was a generous gift from Dr. Peter Sarnow, Stanford University.

Capped RNA used for *in vitro* translation and RNA transfections was *in vitro* transcribed using the mMessage Machine kit (Ambion) per manufacturer's instructions as described in [16]. Uncapped RNA used for *in vitro* binding assays was *in vitro* transcribed using the MEGAScript kit (Ambion) per manufacturer's instructions.

### Cell Culture/Transfections

C6 and N2a cell lines were purchased from ATCC and cultured in DMEM, 10% fetal bovine serum, and 200 mM L-glutamine. Two micrograms of DNA was transfected into the cells using FuGene6 reagent (Roche). The cells were harvested 24 hours later and lysed using passive cell lysis buffer (Promega). The lysate was then assayed for luciferase activity using the Dual-Luciferase Reporter Assay System (Promega) measured using a Luminoskan luminometer.

Co-transfections were performed as described above using 1.8 µg of RP DNA and 0.2 µg of either the Pactag null vector (based on pACTAG-2) or DNA encoding for a hypophosphorylated version of 4E-BP1 [33]. The 4E-BP1 and pACTAG-2 constructs were a generous gift from Dr. Nahum Sonenberg, McGill University.

RNA transfections using 2 µg of RNA were performed using the RNA Transmessenger kit (Qiagen) per manufacturer's instructions. The cells were incubated for seven hours after transfection, harvested, and assayed for luciferase activity.

SH-SY5Y cells were purchased from ATCC and cultured in DMEM, 10% fetal bovine serum, and 200 mM L-glutamine. Cells were plated four days prior to transfection and differentiated by treating with 2 µM retinoic acid. Upon transfection, the media was replaced with DMEM. Three hours into the transfection, the media was exchanged and retinoic acid or the carrier DMSO was added to the cells for the remainder of the incubation.

### PTB1 Purification

Rosetta cells (Novagen) were transformed with the plasmid PGEX-2TK-PTB1, encoding GST-tagged full-length human PTB1. The PTB1 construct was a generous gift from M. A. Garcia-Blanco. Bacteria were grown in 2XYT media to a density 0.35 ODU_595nm_ at which time expression of GST-1 was induced with 0.2 mM IPTG. After 18 hrs of expression at 25°C, the cells were collected by centrifugation and resuspended in lysis buffer (50 mM Tris-HCl pH 8.0, 0.25 M NaCl, 1 mM TCEP, 1 mM EDTA). After lysis by sonication, the NaCl concentration was raised to 1 M and the lysate clarified by centrifugation. The resulting supernatant was applied to glutathione-coupled agarose (GE-Healthcare), and the column was washed with lysis buffer to remove the excess NaCl. To liberate PTB1, thrombin was added and the column was incubated at 25°C for 3 hours with gentle agitation followed by elution of the PTB1. Glycerol was added to the PTB1 containing fractions to a final concentration of ∼10% (v/v). Afterwards, the fractions were concentrated. The PTB1 was further purified by size exclusion chromatography on a Sup200 column (GE Healthcare) in lysis buffer. To remove any bound nucleic acids from PTB1, the protein was bound to a heparin column (GE Healthcare) in lysis buffer, and eluted with a 0.25–2 M NaCl linear gradient. The PTB1 containing fractions were concentrated, dialyzed against the storage buffer (50 mM Tris-HCl pH 8.0, 0.25 M NaCl, 1 mM TCEP, 1 mM EDTA, 20% glycerol (v/v)), and stored at 4°C.

### *In Vitro* Translation

One microgram of *Photinus* luciferase monocistronic mRNA was added to rabbit reticulosyte lysate (RRL) (Red Nova, Novagen) in the presence of cap analog (Ambion). Following a 1 hour incubation at 30°C, the sample was assayed for *Photinus* luciferase activity.

For the *in vitro* translation assays involving PTB1, 1 µg of dicistronic mRNA was added to RRL (Promega) in the presence of 0.4 µg of PTB1 protein. The lysates were incubated for 1 hour at 30°C and then the samples were assayed for luciferase activity as described above.

### PTB1 Binding Assay

Approximately 2 pmol of radiolabeled RNA (500 cpm) was combined with increasing amounts of recombinant PTB1 protein in protein binding buffer (10 mM Tris-HCl pH 8, 100 mM KCl, 2.5 mM MgCl2, 5% glycerol (v/v)) [53] in triplicate. The reaction was incubated for 10 minutes at 37°C and then passed through a dot blot apparatus containing a sandwich of nitrocellulose and charged nylon membranes. A ratio of the fraction of bound RNA to the total RNA was calculated and plotted against the PTB1 concentration. The curve was fit using the Langmuir formula [(m0*m1/(M0+m2)+m3);m1 = .9;m2 = 1e−9;m3 = .001] to determine the dissociation constant.

### siRNA Transfection

SH-SY5Y cells were treated for two days with either DMSO (mock) or 2 µM retinoic acid to induce differentiation. The cells were incubated with 300 µM siRNA (Dharmacon) and Dharmafect 1 reagent (Dharmacon). After 24 hours, the siRNA and media were aspirated off and fresh media was applied to the cells for an additional 24 hours. The cells were treated with DMSO or 2 µM retinoic acid for an additional 48 hours. At this stage, the cells were either harvested for Western blot analysis or transfected with RNA as described above.
